# The fitness cost of horizontally transferred and mutational antimicrobial resistance in *Escherichia coli*

**DOI:** 10.3389/fmicb.2023.1186920

**Published:** 2023-06-30

**Authors:** Marie Vanacker, Natacha Lenuzza, Jean-Philippe Rasigade

**Affiliations:** ^1^CIRI, Centre International de Recherche en Infectiologie, Université de Lyon, Inserm U1111, Université Claude Bernard Lyon 1, CNRS, UMR5308, ENS de Lyon, Lyon, France; ^2^Institut des Agents Infectieux, Hospices Civils de Lyon, Lyon, France

**Keywords:** conjugation, plasmid, mutation, relative fitness, compensatory evolution

## Abstract

Antimicrobial resistance (AMR) in bacteria implies a tradeoff between the benefit of resistance under antimicrobial selection pressure and the incurred fitness cost in the absence of antimicrobials. The fitness cost of a resistance determinant is expected to depend on its genetic support, such as a chromosomal mutation or a plasmid acquisition, and on its impact on cell metabolism, such as an alteration in an essential metabolic pathway or the production of a new enzyme. To provide a global picture of the factors that influence AMR fitness cost, we conducted a systematic review and meta-analysis focused on a single species, *Escherichia coli*. By combining results from 46 high-quality studies in a multilevel meta-analysis framework, we find that the fitness cost of AMR is smaller when provided by horizontally transferable genes such as those encoding beta-lactamases, compared to mutations in core genes such as those involved in fluoroquinolone and rifampicin resistance. We observe that the accumulation of acquired AMR genes imposes a much smaller burden on the host cell than the accumulation of AMR mutations, and we provide quantitative estimates of the additional cost of a new gene or mutation. These findings highlight that gene acquisition is more efficient than the accumulation of mutations to evolve multidrug resistance, which can contribute to the observed dominance of horizontally transferred genes in the current AMR epidemic.

## 1. Introduction

Antimicrobial resistance (AMR) increases at an alarming rate worldwide, imposing a considerable burden to health systems and menacing the safety of modern medical procedures (Nadeem et al., 2020). AMR has developed against virtually all antibiotics in clinical use (Andersson and Levin, 1999; Levin et al., 2000; Acar and Röstel, 2001; Pope et al., 2010; Nadeem et al., 2020). AMR genes are present in most environments including livestock, sewage, or rivers (Jian et al., 2021), and many genes can access new bacterial species through horizontal transfer, enhancing their dissemination potential across ecological niches. For instance, Pu et al. (2019) have highlighted how carbapenem resistance in *Escherichia coli* provided by the *bla*_NDM_ AMR gene can be shared across humans, dogs, flies, and wild birds in farms. In antibiotic-free environments, however, resistant bacteria are expected to incur a fitness cost (practically, a reduced growth rate) and to be eventually outcompeted by their susceptible, more fit counterparts. This fitness cost may result from the alteration of an enzyme by mutation, the disruption of a metabolic pathway following gene loss or inactivation, or the additional energy required for the overexpression of a gene or the expression and replication of a new gene acquired through horizontal transfer.

The biology and ecology of AMR emergence differ fundamentally depending on whether AMR results from the mutation of a chromosomal gene (hereafter, AMR mutation) or the acquisition of a transferable AMR gene (Acar and Röstel, 2001). AMR mutations typically alter essential enzymes while transferable AMR genes typically provide new enzymes that may or may not interfere with cell metabolism. From an ecological standpoint, AMR mutations and transferable AMR genes also differ because the unit of selection of the vertically inherited mutations is the bacterial cell and its offspring, while transferable genes can emancipate from their host and become themselves the unit of selection. Therefore, the impact of fitness cost on the persistence of transferable AMR genes is expected to differ from AMR mutations (Vogwill and MacLean, 2015). This point has practical importance because transferable AMR genes, including those encoding extended spectrum beta-lactamases, carbapenemases, or aminoglycoside modifying enzymes are the main drivers of the current AMR epidemic in enterobacteria, while AMR mutations are less problematic. It is still unclear, however, whether the fitness cost of AMR depends on the transfer potential of the AMR determinant. Most comparative studies of AMR fitness cost have either focused on fitness variations across AMR mechanisms, mutations, drug families or bacterial species, rather than on the difference between transferable and non-transferable resistance.

To fill this knowledge gap, we conducted a comparative meta-analysis of the fitness cost of resistance, with a focus on the transferable nature of AMR determinants. We focused on a single, well-studied species, *E. coli*, to ease interpretation of the results by avoiding biasing fitness evaluation across multiple host species. Using a multilevel meta-analysis framework, we examined whether the fitness cost of AMR, from a single determinant to an accumulation of many genes or mutations, differs when provided by horizontally transferable genes such as those encoding beta-lactamases, comparable to mutations in core genes such as those involved in fluoroquinolone and rifampicin resistance.

## 2. Materials and methods

### 2.1. Estimation of the relative fitness associated with AMR determinants

This is a systematic review and meta-analysis of the relative fitness of AMR determinants in *E. coli*. Several procedures exist to estimate relative fitness, mostly based on competition assays between a resistant strain and its susceptible, isogenic counterpart. We retained three different estimations of relative fitness, briefly described below.

In the first estimation method described by Lenski et al. (1991), the relative fitness $W_{r}$ (Equation 1) is the ratio of the Malthusian parameters m (or exponential growth rate) of a resistant mutant ( $m_{R}$ ) and a susceptible strain ( $m_{S}$ ). The parameter m is usually estimated experimentally as the logarithm of the ratio of the final population size $N^{t}$ (after t epochs) on the initial population size $N^{0}$ , by solving the exponential growth equation $N^{t}=N^{0}e^{mt}$ . The ratio of Malthusian parameters can be written:


(1) Wr=mRmS≈lnNRtNR0lnNStNS0

Remark that this equation does not explicitly take t into account, and the estimates may vary with the duration of the growth assay.

The second estimation method (Lenski, 1991) explicitly takes the duration of the competition assay into account by measuring population sizes $N_{R}$ and $N_{S}$ of the resistant and susceptible strains, respectively, at different time points and by regressing the logarithm of their ratio, ln(NRNS) , over time. The relative fitness $W_{s}$ is defined as the complement of the regression slope s , $W_{s}=1-s$ .

The third estimation method (Dykhuizen and Hartl, 1983; Dean and Dykhuizen, 1988; Dykhuizen, 1990) is based on the increase per time unit of the difference between the Malthusian parameters of the resistant and the susceptible strain:


(2) Wt=1+mR−mSt≈1+1t(ln(NRtNR0)−ln(NStNS0))

The three estimators $W_{r}$ (Malthusian ratio), $W_{s}$ (regression slope), and $W_{t}$ (Malthusian difference per time unit) are collectively referred to as the relative fitness W . A relative fitness W=1 indicates an absence of effect of resistance, W<1 indicates a fitness cost, and W>1 indicates a fitness advantage of the resistant strain. Although the qualitative interpretation of neutral, reduced, or increased fitness is common to all three methods of estimations, these methods differ quantitatively. $W_{r}$ is a dimensionless ratio that lacks any direct interpretation and that can only be used to compare experiments of the same duration t , typically 24 h, as this duration is implicit in Eq. 1. $W_{s}$ has a more direct interpretation because it reflects the relative increase of population size through time, however, it is expressed in logarithmic units and involves a complement that obfuscates its biological meaning. Finally, $W_{t}$ has a similar interpretation as $W_{s}$ because it represents a relative increase per unit of time, using the Malthusian parameter in place of the logarithm of population size used in $W_{s}$ . It should be noted that neither $W_{r}$ , $W_{s}$ , or $W_{t}$ are meaningful quantitative representations of the fitness because they involve either ratios of logarithms (Eq. 1), which are dimensionless, or slopes of logarithms. More interpretable quantities may be derived from, for instance, the ratio of the doubling time of the resistant and competitor strains. However, such quantities are not in common use and could not be recovered from the relative fitness data $W_{r}$ , $W_{s}$ , or $W_{t}$ , reported in the included studies.

The biological impact of the relative fitness can be illustrated more intuitively by computing the number of generations $t=\log_{2}1000/\left(1-W_{R}\right)$ after which the susceptible variant becomes 1,000x more prevalent than the resistant variant (the relation for t is easily derived from Eq. 1). A relative fitness $W_{R}=0.9$ yields t=99.7 , which means that ~100 generations (33 h for *E. coli*, assuming a 20 min doubling time) are sufficient to virtually eliminate the resistant variant. Hence, a relative fitness of 0.9 may be considered a very strong fitness cost. For $W_{R}=0.99$ , the susceptible variant becomes 1,000x more prevalent than the resistance variant after t≈1,000 generations, or 2 weeks for *E. coli*. Hence, a relative fitness of 0.99 may be considered a moderate fitness cost. The relation for t also illustrates that a relative fitness very close to 1, which may be difficult to estimate experimentally, can still have a substantial impact on the bacterial population over months or years of competition.

### 2.2. Literature search and inclusion criteria

We searched the PubMed database using terms ‘fitness’ and ‘*Escherichia coli*’ or ‘*E. coli*’ and ‘antibiotic resistance’ or ‘antimicrobial resistance’ or ‘drug resistance’. Search results were limited to peer-reviewed studies in English available online by the 29th September 2022. No start date was specified.

To be included, studies had to report relative fitness findings numerically in the text, in a table or a figure. Relative fitness measurements should meet the following criteria. The relative fitness had to be measured in competition assays in an approximate proportion of 1:1 between a resistant strain (mutant) and a wild-type (susceptible or ancestral) strain only differing by the absence of resistance. Studies in which the data were reported in an unstandardized protocol without competition with a control strain were excluded. Studies comparing two resistant strains were also excluded. The competition assays had to be conducted at 37°C in a stable antibiotic free environment. Experiments involving a modification of environmental conditions were excluded. Finally, relative fitness had to be estimated using one of the three methods $W_{r}$ , $W_{s}$ or $W_{t}$ described above. Estimations based on relative growth rates, competitive indices or uncommon methods were excluded.

**Figure 1:**
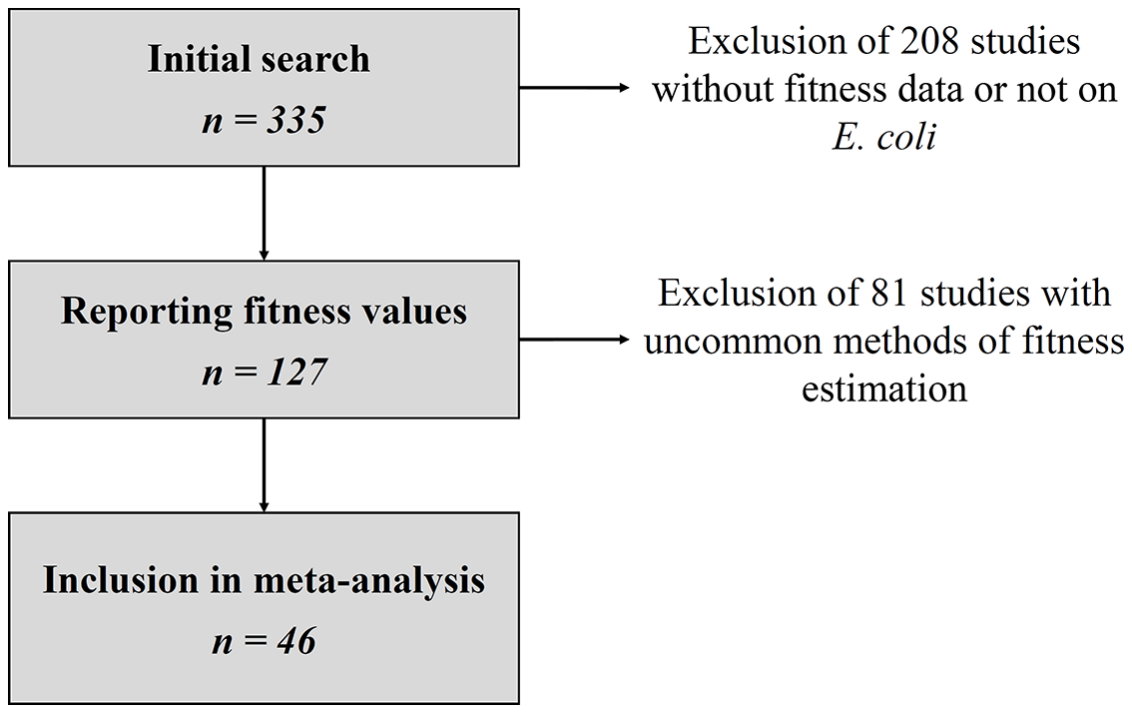
Flowchart of study selection. Details of eligible studies and reasons for exclusion can be found in the Supplementary Data S1.

Of 335 studies matching the search criteria, 127 reported relative fitness values and 46 were included in the final dataset (Figure 1), representing a total of 783 resistant strains. Details of all eligible studies and reasons for exclusion where applicable are given in the Supplementary Data S1. For each resistant strain and competition assay included in the final analysis, we collected the duration of the assay, the culture medium, the relative fitness estimation method, the mean and standard error of the relative fitness, the number of experiment replicates, the nature of the susceptible strain (either isogenic, or ancestral), the nature of its differences relative to the resistant strain (such as plasmid loss, mutation) and the number of AMR genes or mutations in the resistant strain. Detailed datasets can be found in the Supplementary Data S2, S3. Out of the 46 studies, 22 used the $W_{r}$ estimator of the relative fitness, 11 used the $W_{s}$ estimator, and 13 used the $W_{t}$ estimator.

### 2.3. Characteristics of AMR determinants

Genetic determinants of AMR in each resistant strain were classified as either acquired AMR genes, such as *bla*Oxa48, when harbored on mobile genetic elements, or AMR mutations and indels in non-transferable genes such as *rpo*B, referred to as mutated AMR genes. For each acquired AMR gene or mutation, the class, family, and mechanism of action of the targeted antibiotic was identified from the literature (McArthur et al., 2013; Poirel et al., 2018). Genes whose acquisition or mutation confer resistance to several drug families, such as *acr*B, were also identified as such (McArthur et al., 2013; Poirel et al., 2018; Wang et al., 2019; Brandis et al., 2021; Praski Alzrigat et al., 2021; Rajer and Sandegren, 2022). As acquired AMR genes may be harbored by plasmids, transposons, integrated plasmids or transposons integrated in plasmids, we did not distinguish between plasmid- and transposon-borne genes in our analyses to avoid ambiguity. When acquired AMR genes were explicitly reported as plasmid-borne, we collected the size of plasmid, the number of harbored AMR genes and the incompatibility group.

### 2.4. Statistical analysis

A multilevel meta-analysis of relative fitness, taking into account three levels of analysis, was conducted as described by Harrer et al. (2022):

- The first level is the level of interest for the analysis, comparing groups of resistant strains based on the relative fitness average and standard error. Several strains were thus recorded for each study.
- The second level captures intra-study variation.
- The third level captures inter-study variation and the pooling of the aggregated cluster effects leading to the overall effect.

The overall effect size ${\hat{\theta }}_{ij}$ is the effect of the strain *i* nested in the study *j* as described in Equation 3, where μ is the overall mean population effect, δ_(2)ij_ the intra-study heterogeneity at level 2, δ_(3)j_ the inter-study heterogeneity at level 3, and ε_(1)ij_ the sampling error of strains estimated as the standard deviation of relative fitness.


(3) $${\hat{\theta }}_{ij}=\mu +\delta_{\left(2\right)ij}+\delta_{\left(3\right)j}+\varepsilon_{\left(1\right)ij}\;$$

The share of variance not attributable to sampling error in intra and inter-study heterogeneity was calculated using the *I*^2^ statistic (Higgins and Thompson, 2002). The significance threshold was set at 5% without correction for multiple testing, in line with the exploratory nature of the analysis. To estimate the additional fitness cost associated with the accumulation of AMR mutations or acquired AMR genes, meta-regression models were constructed using the relative fitness as the response variable and the number of mutated or acquired AMR genes per strain, or the number of drug resistance families (e.g., beta-lactams or fluoroquinolones) as a covariate. Meta-regression analyses were conducted on strains containing either only AMR mutations or only acquired AMR genes to avoid mixing the effect of gene mutation and acquisition in the models. Where applicable, meta-regression models used a fixed intercept of 1 (neutral fitness) to account for the fact that the absence of AMR mutation, gene or resistance should yield the same fitness as the comparator strain. All analyses were performed using R version 4.1 (R Core Team, 2022) with additional packages ‘metafor’ (Viechtbauer, 2010), ‘meta’ (Balduzzi et al., 2019), ‘forestploter’ (Dayimu, 2022), and ‘dmetar’ (Harrer et al., 2019).

## 3. Results

### 3.1. Influence of experimental conditions on the estimation of relative fitness

We searched for sources of heterogeneity in the estimation of relative fitness across the 46 studies included in the analysis, totaling 783 resistant *E. coli* strains. A significant inter-study heterogeneity was found, based on *I*^2^ test (Supplementary Figure S1). A funnel plot analysis showed a high asymmetry between the mean standardized difference and the between-study standard errors of relative fitness, suggesting the presence of publication bias. This publication bias was also confirmed by Egger’s test with a value of *p* < 0.001(Supplementary Figure S2). The decomposition of variance in multi-level analysis (Supplementary Figure S3) attributed 32% of the overall variance to between-study variation (analysis level 3), 68% to within-study variation (level 2) and 0% to strain-level variation (level 1). Hence, heterogeneity was most concentrated at the within-study level. Both the culture medium and the duration of competition assays had a moderate influence on relative fitness (Supplementary Figure S4A). Most assays used Luria-Bertani medium (*n* = 501 strains out of 783, 63.4%) over 24 h (*n* = 517 strains, 66.0%). Relative fitness estimates decreased significantly with the duration of competition assays (*p* = 0.035; Supplementary Figure S4B), suggesting that longer experiments potentialize or better reveal the fitness cost.

**Figure 2:**
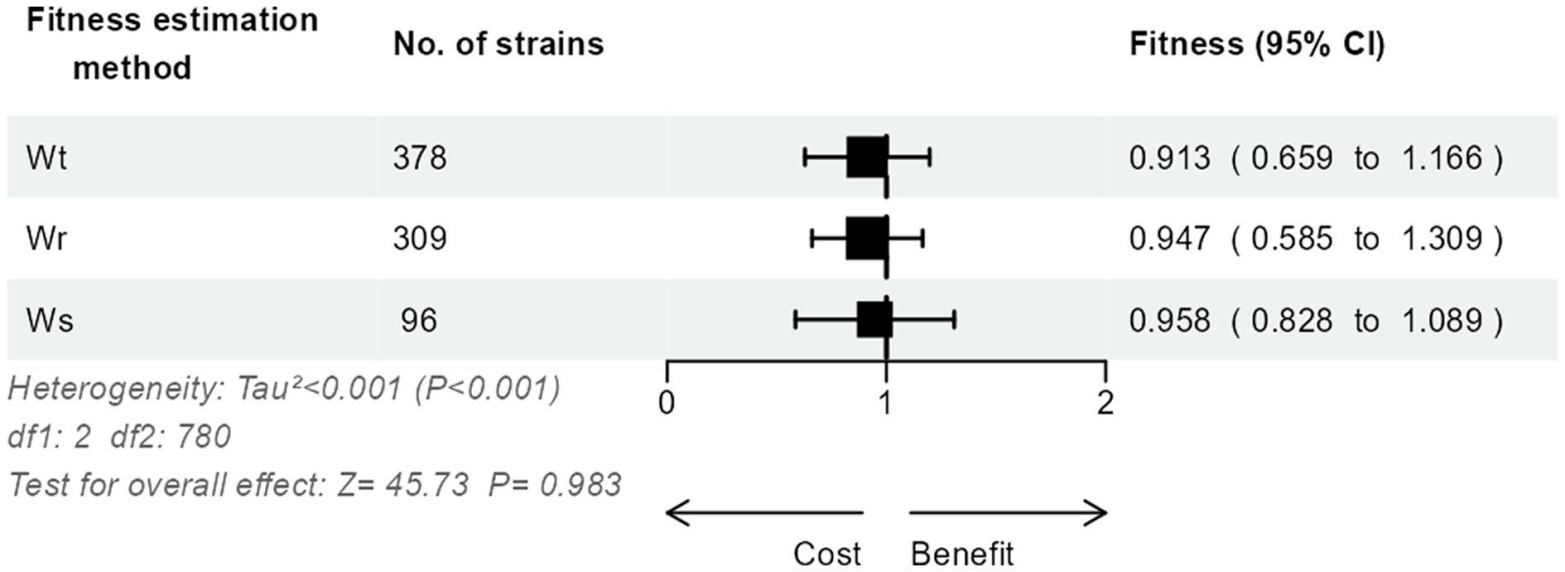
Three-level meta-analysis of the impact of the estimation method on the results of relative fitness in competition experiments of 783 *E. coli* strains from 46 studies. Shown are forest plots of the relative fitness estimated using either the Malthusian difference per time unit $W_{t}$ , the Malthusian ratio $W_{r}$ , or the regression slope method $W_{s}$ . Square markers denote effect size, marker size is proportional to the group weight in the overall effect, and error bars denote the 95% confidence interval. No significant overall effect of the estimation method was observed.

The relative fitness of the 783 strains had been calculated using either the $W_{t}$ method (Malthusian difference per time unit; *n* = 378, 48.3%), the $W_{r}$ method (Malthusian ratio; *n* = 309, 39.5%), or the $W_{s}$ method (regression slope; *n* = 96, 12.3%). Interestingly, we could not detect a significant impact of the estimation method on the relative fitness (*p* = 0.98; Figure 2).

Using goodness-of-fit analyses, we observed that a three-level meta-analysis model, taking inter-study variation into account, outperformed a simpler, two-level model in terms of Akaike information criterion and likelihood ratio test (Table 1). In line with this observation, we used three-level models in the subsequent analyses.

**Table 1:** Model comparison between a three-level model (inter-study variance) and a two-level model (intra-study variance) for the meta-analysis of relative fitness in 783 *E. coli* strains from 46 studies.

	Degree of freedom	AIC	Log-likehood	Likehood ratio test (LRT)	LRT value of *p*
Three-level model	3	−971	489		
Two-level model	2	−736	370	238	<0.0001

1. The best model is the one with the lowest value of AIC. 2. The best model is the one with the highest value of log-likehood.

### 3.2. Relative fitness comparison of AMR mutations and gene acquisitions

**Figure 3:**
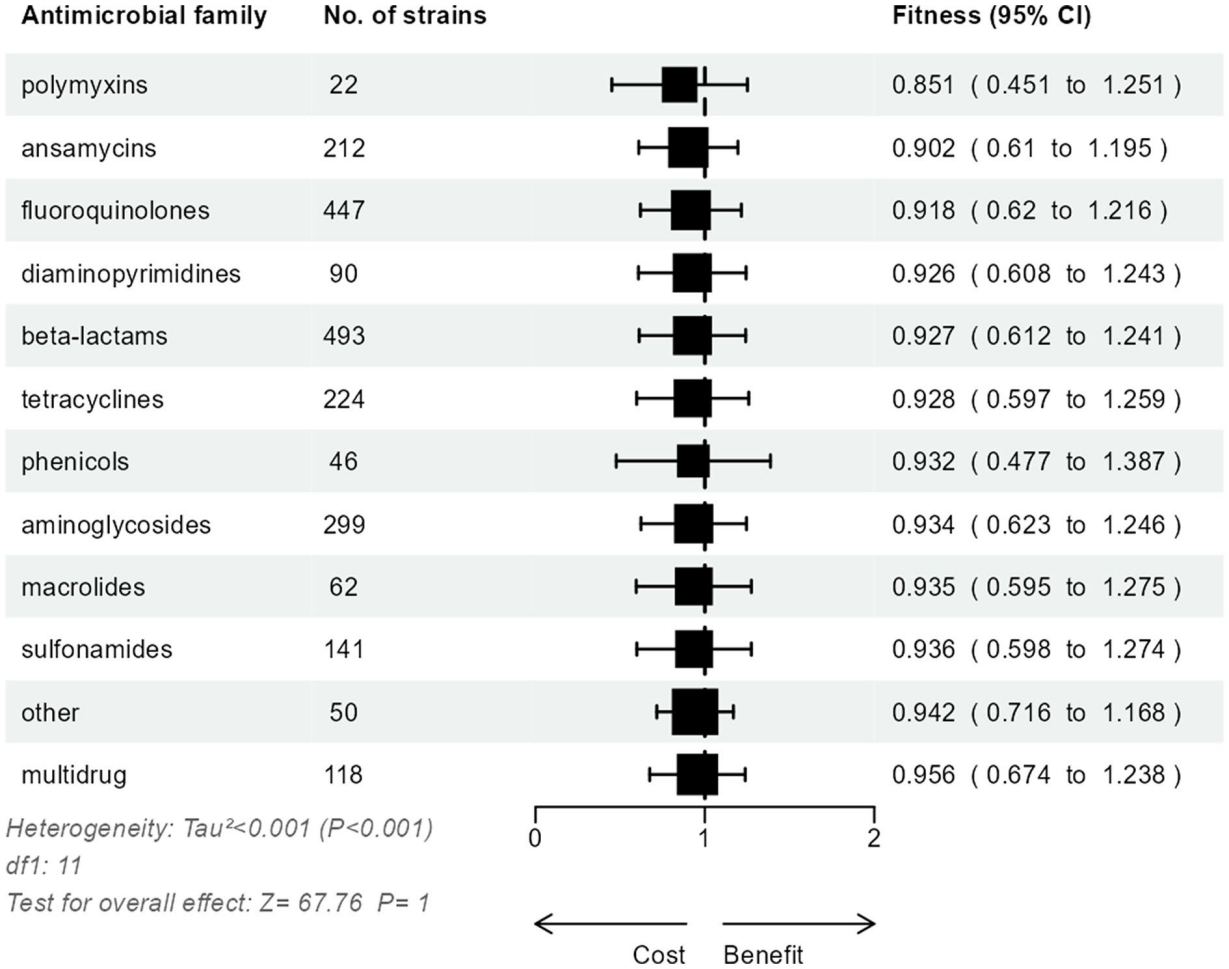
Three-level meta-analysis of the relative fitness of *E. coli* strains (*n* = 783) resistant to various antimicrobial drug families. Square markers denote effect size, marker size is proportional to the group weight in the overall effect, and error bars denote the 95% confidence interval. No significant overall effect of the resistance to specific drug families was observed.

A total of 146 unique AMR genes were studied in relative fitness experiments, of which 78 (53.4%) were acquired and 68 (46.6%) were mutated. One hundred and sixty-three unique mutations were identified among the mutated AMR genes, corresponding to an average of 2.7 mutations per gene (range, 1 to 31). The AMR determinants most commonly studied were *rpo*B and *gyr*A mutations (26.7 and 24.4% of strains, respectively; Table 2) and the most common acquired AMR gene was *bla*_TEM_ (12.3%). One hundred and thirty strains (16.6%) included both mutated and acquired AMR genes. Strains with only mutated AMR genes were resistant to an average of 2.2 drug families, with a maximum of 5 drug families in a strain carrying AMR mutations in 11 different genes (Brandis et al., 2021). Strains with only acquired AMR genes carried resistance to 4.3 drug families, with a maximum of 8 drug families in a strain carrying plasmids each containing 10–14 AMR genes (Rajer and Sandegren, 2022). A meta-analysis model comparing the relative fitness of strains harboring resistance to each drug family did not show significant differences (*p* = 1 for the overall effect; Figure 3) between groups, although a trend toward a lesser relative fitness of polymixin-resistant strains (fitness 0.85) was observed compared to other resistances with relative fitness >0.90.

**Table 2:** The mutated or acquired AMR genes present in >5% of 783 resistant *E. coli* strains from 46 studies of relative fitness.

Gene	No. of strains (%), *n*=783^1^	Genetic support	AMR family	References
*aad*A5	49 (6.3%)	Acquisition	Aminoglycosides	McArthur et al. (2013), Poirel et al. (2018), and Rajer and Sandegren (2022)
*acr*R	58 (7.4%)	Mutation	Multidrug	Praski Alzrigat et al. (2021)
*aph*(3″)-Ib	50 (6.4%)	Acquisition	Aminoglycosides	McArthur et al. (2013), Poirel et al. (2018), and Tang et al. (2022)
*aph*(6)-Id	58 (7.4%)	Acquisition	Aminoglycosides	McArthur et al. (2013), Poirel et al. (2018), and Rajer and Sandegren (2022)
*bla*CTXM-15	52 (6.6%)	Acquisition	Beta-lactams	Poirel et al. (2018) and Alonso-del Valle et al. (2021)
*bla*OXA	92 (11.7%)	Acquisition	Beta-lactams	Silva et al. (2011) and Poirel et al. (2018)
*bla*OXA-1	45 (5.7%)	Acquisition	Beta-lactams	Poirel et al. (2018), Palkovicova et al. (2022), and Rajer and Sandegren (2022)
*bla*TEM	96 (12.3%)	Acquisition	Beta-lactams	Poirel et al. (2018) and Cai et al. (2021)
*bla*TEM-1	62 (7.9%)	Acquisition	Beta-lactams	Dahlberg and Chao (2003) and Poirel et al. (2018)
*bla*TEM-1B	46 (5.9%)	Acquisition	Beta-lactams	Poirel et al. (2018), Alonso-del Valle et al. (2021), Rajer and Sandegren (2022), and Tang et al. (2022)
*dfr*A17	49 (6.3%)	Acquisition	Diaminopyrimidines	Poirel et al. (2018), Brandis et al. (2021), and Tang et al. (2022)
*gyr*A	191 (24.4%)	Mutation	Fluoroquinolones	Marcusson et al. (2009), Huseby et al. (2017), Wang et al. (2017), and Poirel et al. (2018)
*gyr*B	62 (7.9%)	Mutation	Fluoroquinolones	Wang et al. (2017) and Poirel et al. (2018)
*mar*R	108 (13.8%)	Mutation	Fluoroquinolones, beta-lactams	Marcusson et al. (2009), Huseby et al. (2017), Wang et al. (2017), Brandis et al. (2021), and Praski Alzrigat et al. (2021)
*mph*(A)	62 (7.9%)	Acquisition	Macrolides	Liu et al. (2021) and Rajer and Sandegren (2022)
*par*C	58 (7.4%)	Mutation	Fluoroquinolones	Marcusson et al. (2009) and Poirel et al. (2018)
*rpo*B	209 (26.7%)	Mutation	Fluoroquinolones, beta-lactams, ansamycins	Angst and Hall (2013), Durão et al. (2015), and Brandis et al. (2021)
*rps*L	118 (15.1%)	Mutation	Aminoglycosides	Angst and Hall (2013) and Durão et al. (2015)
*sul*1	84 (10.7%)	Acquisition	Sulfonamides	Enne et al. (2005) and Poirel et al. (2018)
*sul*2	68 (8.7%)	Acquisition	Sulfonamides	Enne (2004) and Poirel et al. (2018)
*tet*(A)	75 (9.6%)	Acquisition	Tetracyclines	Poirel et al. (2018), Alonso-del Valle et al. (2021), and Zhang et al. (2022)
*tet*(X)	79 (10.1%)	Acquisition	Tetracyclines	Poirel et al. (2018), Rajer and Sandegren (2022), and Tang et al. (2022)

1 Total % higher than 100 because strains can contain multiple AMR genes.

**Figure 4:**
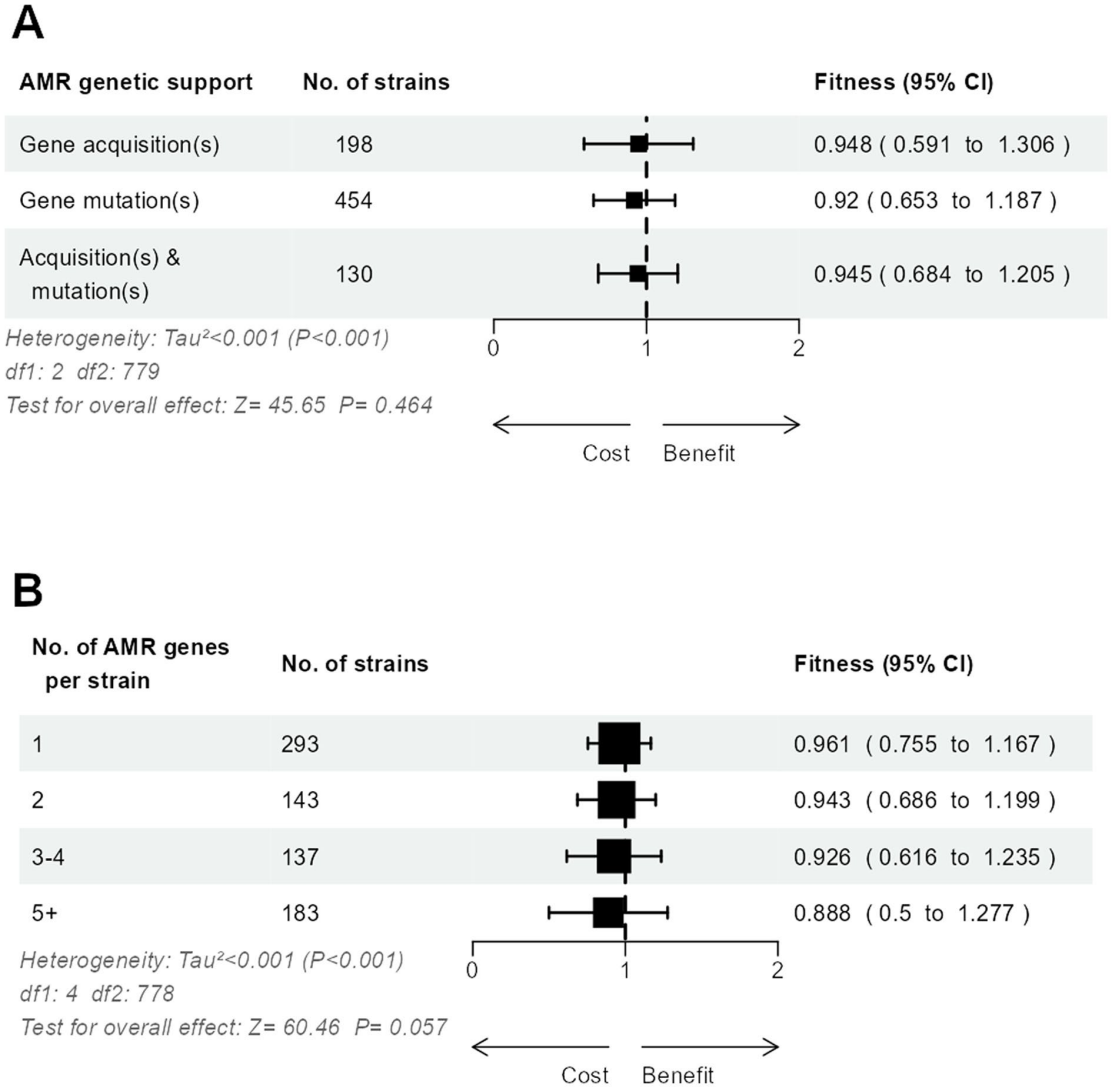
Three-level meta-analysis of the relative fitness of *E. coli* strains (n = 783) with mutated and/or acquired AMR genes **(A)**, and according to the number of mutated and acquired AMR genes per strain **(B)**. Shown are forest plots in which square markers denote effect size, marker size is proportional to the group weight in the overall effect, and error bars denote the 95% confidence interval.

The relative fitness did not differ significantly between strains with only mutated AMR genes, only acquired AMR genes, or both (Figure 4A). When strains were grouped according to the number of AMR genes, either mutated or acquired, we observed a possible trend toward a lesser relative fitness in strains accumulating more resistance, from 0.96 in strains with only one AMR gene to 0.89 in strains with five or more AMR genes (Figure 4B).

**Figure 5:**
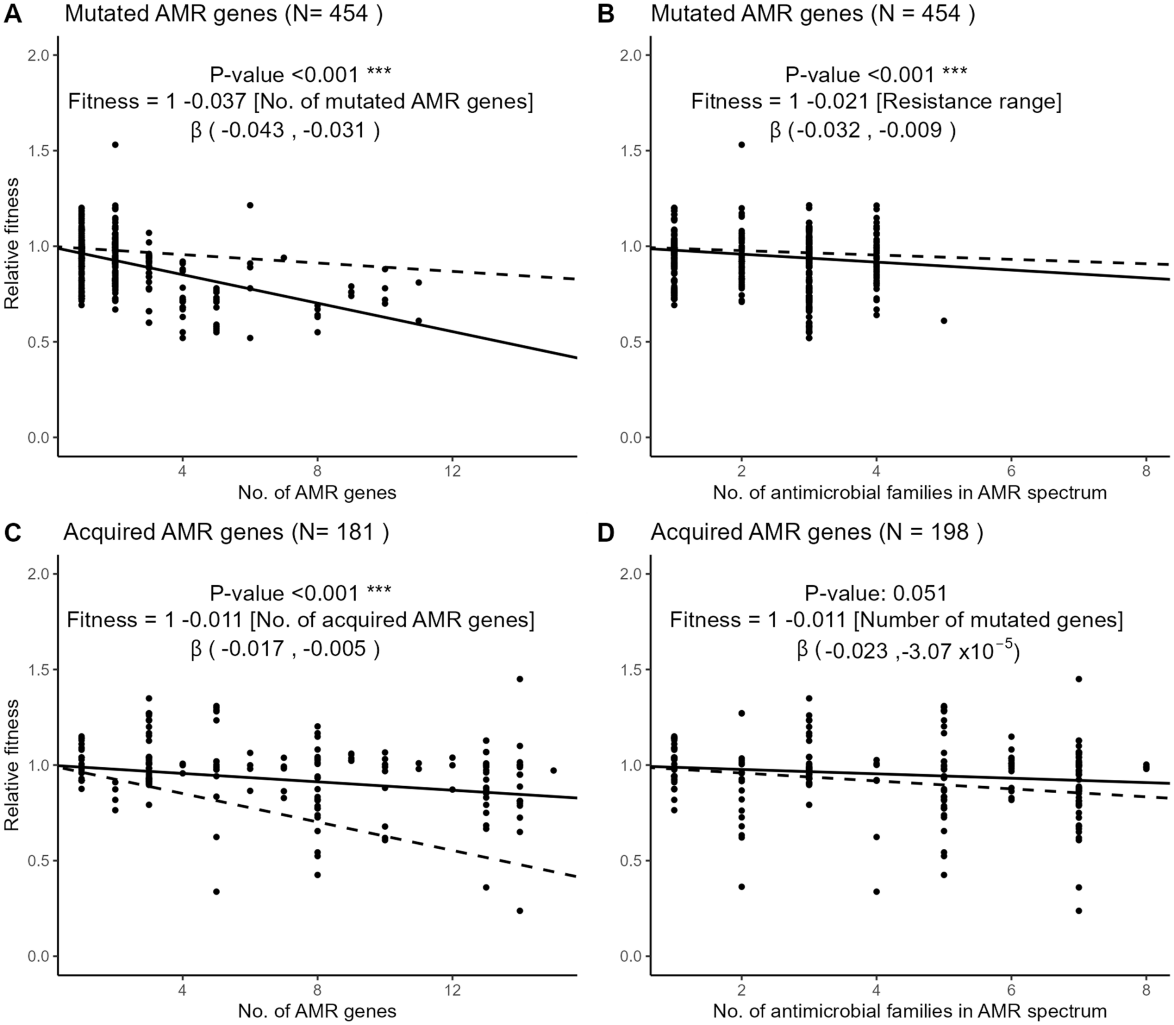
Three-level meta-regressions of the relative fitness of *E. coli* strains with either mutated **(A,B)** or acquired **(C,D)** AMR genes as a function of the number of AMR genes **(A,C)** or resistant drug families **(B,D)** per strain. Shown are meta-regression plots where each point denotes a strain and the solid line denotes the regression slope with a fixed intercept of 1. Dashed lines denote the regression slope in the comparator group, that is, the dashed line in panel **A** (mutated AMR genes) represents the regression slope of panel **C** (acquired AMR genes). The regression coefficient estimates the decrease of relative fitness with each additional AMR gene or resistant drug family. The 95% confidence interval of the β regression coefficient is shown in brackets. Antimicrobial drug families are listed in Figure 3. Sample sizes differ in panels **C,D** because of the exclusion of strains with missing information on either the no. of AMR genes or the no. of drug family resistances.

To examine the fitness impact of gene mutation or acquisition on AMR accumulation, we used meta-regression models to quantify the change of relative fitness with each additional mutated or acquired AMR gene (Figures 5A,C). Each additional mutated AMR gene significantly decreased fitness by 3.7% (95% CI, 3.1 to 4.3%) while each additional AMR gene acquisition decreased fitness by 1.1% (95% CI, 0.5 to 1.7%). Hence, the cost of each additional AMR mutation was more than 3-fold higher than the cost of each additional AMR gene acquisition. Using the same meta-regression method, we observed that the accumulation of resistance to several antimicrobial families was more costly when AMR resulted from gene mutations (Figures 5B,D). Each additional drug family in the resistance spectrum significantly decreased the relative fitness by 2.1% (95% CI 0.9 to 3.2%) in strains with only mutated AMR genes. In strains with only acquired AMR genes, however, the decrease of relative fitness per additional drug family was only 1.1% and not significantly different from zero (95% CI, −0.003 to 2.3%).

### 3.3. Fitness decreases with the size but not the number of AMR plasmids

**Figure 6:**
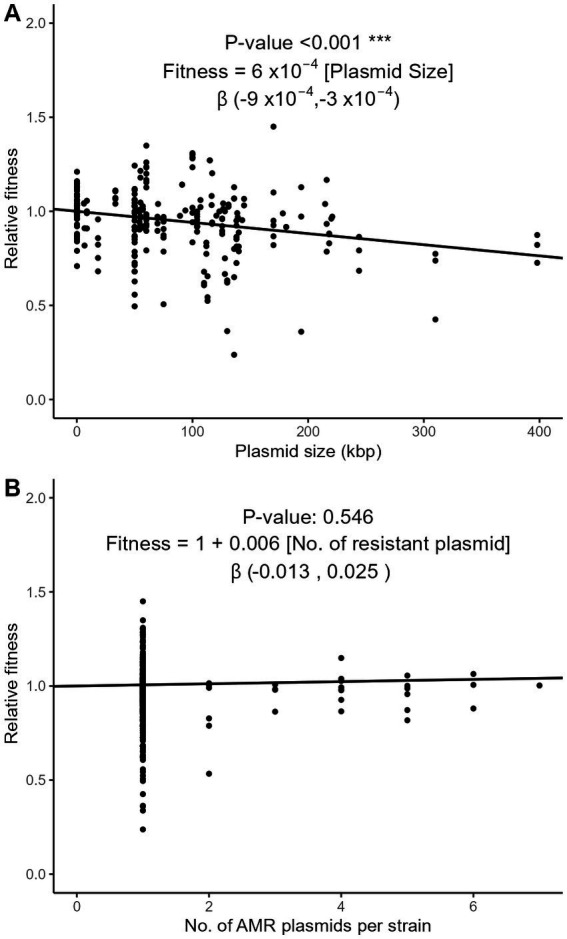
Three-level meta-regression of the relative fitness of *E. coli* strains with plasmid-borne AMR genes based on the size **(A)** and number **(B)** of AMR plasmids. The effect of plasmid size **(A)** was analyzed in the subset of strains harboring exactly one AMR plasmid (*n* = 266). The effect of the number of plasmids per strain **(B)** was analyzed in the strains with at least one AMR plasmid (*n* = 325), with a fixed intercept of 1. The regression coefficient estimates the decrease of relative fitness with each additional kbp in plasmid length **(A)** or each additional AMR plasmid **(B)**. The 95% confidence intervals of the β regression coefficient are shown in brackets.

Plasmids require energy for their own maintenance and replication. We examined whether the relative fitness was influenced by plasmid characteristics such as their size, number per strain, and incompatibility group (Inc). Using the meta-regression approach on the subset of strains with exactly one AMR plasmid (*n* = 266), we observed that plasmid size, that averaged to 83 kbp, significantly correlated with a decrease of fitness cost of 0.06% per kbp (95% CI, 0.03–0.09%; Figure 6A). In strains with one or more plasmids, however, the number of plasmids had no significant effect on relative fitness (Figure 6B). The conjugative nature of the plasmid, compared to non-conjugative plasmids and to plasmids engineered *in vitro* as vectors of AMR genes, had no significant impact on relative fitness (Supplementary Figure S5A). Nine major incompatibility groups of plasmids were represented in our dataset. The relative fitness did not significantly differ between groups (Supplementary Figure S5B), although possible trends were observed. The most frequent incompatibility group was IncF (*n* = 88 strains), with an average relative fitness of 0.928. Strains with IncA/C and IncX plasmids appeared more costly than the other Inc. groups (0.747 and 0.872, respectively), while IncP plasmids seemed to have a lesser impact on fitness (Supplementary Figure S5B).

## 4. Discussion

In this meta-analysis of 783 drug-resistant *E. coli* strains from 46 studies, we observed that the accumulation of AMR resulting from mutations of chromosomal genes entails a 3-fold stronger fitness cost than the accumulation of transferable AMR genes. This phenomenon may contribute to the observed dominance of transferable AMR genes in the current multidrug resistance epidemic in enterobacteria.

The most problematic lineages of multidrug resistant enterobacteria have evolved through the acquisition of horizontally transferred, mainly plasmid-borne, AMR genes rather than the accumulation of AMR mutations (Acar and Röstel, 2001). Previous work has repeatedly identified that mutational AMR is more costly than AMR gene acquisition (Vogwill and MacLean, 2015), however, the impact of the accumulation of AMR determinants through both mechanisms had never been compared. Here, we find that even though the accumulation of acquired AMR genes entails a significant cost, this cost is much reduced compared to the accumulation of AMR mutations (Figure 5). Thus, the evolutionary pathway to multidrug resistance may be strongly facilitated by the availability of transferable AMR genes in the environment, at least in species with HGT capabilities. In contrast with gene acquisition, mutational resistance is available to virtually all species in all environments. This may explain why mutational resistance prevails in species without HGT capabilities such as *Mycobacterium tuberculosis* (Merker et al., 2018), or in confined environments with a limited supply of mobile genetic elements, such as chronic lung infections with *Pseudomonas aeruginosa* (López-Causapé et al., 2018).

Interestingly, the number of AMR genes as well as the size of AMR plasmids were the dominant factors influencing the relative fitness in our analyses. Other plasmid characteristics such as their conjugative or mobilizable nature, or their incompatibility group, had a comparatively negligible impact (Supplementary Figure S5). This suggests that the AMR genes themselves and their number, rather than their plasmid vehicle, are the main source of fitness cost. This is important as the number of AMR genes present in each plasmid may vary considerably across mobilizable, non-mobilizable or conjugative plasmids (in average 2.7, 3.5 and 4.7 AMR genes per plasmid, respectively; Che et al. (2021)). As previously discussed by Vogwill and McLean in their multi-species meta-analysis of fitness cost (Vogwill and MacLean, 2015), AMR genes may be more recent in evolution than other plasmid genes and this shorter adaptation time may contribute to a comparatively higher cost of AMR genes. This hypothesis is supported by recent data suggesting that the fitness cost of plasmid genes results from conflicts with other genes that can be quickly alleviated by fitness-cost compensatory mutations (Yano et al., 2016; Hall et al., 2021). Of note, several of our findings are in contrast with those of the Vogwill and McLean study. They found a significant difference in the relative fitness of mutational and acquired AMR independent of AMR accumulation, while in our study the difference was only revealed by AMR accumulation (Figures 4, 5). In addition, the significant impact of plasmid size on relative fitness that we observed in isolates carrying a single AMR plasmid (Figure 6A) was not observed in their analysis. We speculate that, beyond the influence of new data accumulated since the Vogwill and McLean study, in which *E. coli* accounted for less than 30% of the data (Supplementary Table S1), our focusing on a single species in a multi-level meta-analysis framework may explain these differences.

Our study did not address the possible reasons for the lesser fitness cost of transferable AMR compared to mutational AMR, yet, recent data suggest that compensatory evolution may play a key role in this lesser fitness cost. Indeed, the fitness cost of AMR is a transient property that may decline if a sustained selection pressure enables compensatory evolution to modulate fitness cost. Compensatory evolution, by which additional genomic alterations reduce the fitness cost of AMR but not its resistance level, may be involved in both mutation- and HGT-driven resistance (Durão et al., 2018; Yang et al., 2020; Hall et al., 2021; Patel and Matange, 2021). We speculate that compensatory evolution, in combination with the additional plasticity provided by plasmid-borne AMR, may contribute to preserve both the fitness and the resistance level provided by transferable AMR genes. Indeed, in antibiotic-free environments, the reversion to a more fit, susceptible phenotype is not only driven by the outgrowth of resistant variants by their susceptible ancestors (if they survived), but also by the *de novo* emergence of susceptible variants in the resistant population. Plasmid loss provides an efficient means for this emergence, while the reversion of mutational resistance follows a more complex pathway in which additional mutations contribute to reducing both the fitness cost and the resistance level conveyed by the initial AMR mutation (Dunai et al., 2019).

We acknowledge several limitations to our study. The fitness cost of AMR is difficult to measure in controlled conditions and the experimental procedures are not standardized, which can introduce noise and bias in meta-analysis approaches. For instance, a meta-analysis by Melnyk et al. (2015) found that the relative fitness assessed by the $W_{r}$ method (ratio of Malthusian parameters) exceeded by a factor of 1.7 those assessed by the $W_{t}$ method (Dykhuizen and Hartl, 1983; Dean and Dykhuizen, 1988; Dykhuizen, 1990). Yet, in our study this bias did not translate into a measurable difference of fitness estimations across studies, suggesting that other sources of variation outweighed the influence of the estimation method for biologically relevant values of relative fitness. Indeed, variations of culture media or competition assay duration were common in our dataset, but the observed influence on the estimation of fitness cost was at most moderate (Supplementary Figure S4), enabling for the joint analysis of those experiments in a multi-level analysis framework. Based on previous findings (Melnyk et al., 2015), a major source of variation in relative fitness is the bacterial species itself. Here, we avoided this source of noise by focusing on a single species, although we cannot exclude an unseen influence of the strains’ genetic backgrounds, which were not available for analysis. In addition, our results are focused on the *E. coli* species and further research is needed to determine whether our conclusions hold in other HGT-capable species. Virtually all acquired AMR genes in our analysis were plasmid-borne and our results may not hold for other HGT vehicles such as bacteriophages. Bacteriophages have been suspected to contribute to AMR HGT in several species including *E. coli* (Billard-Pomares et al., 2014), however their relative contribution to AMR is still unclear, considering that transduction rates are several orders of magnitude lower than conjugation rates (Nazarian et al., 2018). Finally, data remain scarce regarding the fitness impact of AMR *in vivo*, and it is still unclear whether conclusions from *in vitro* experiments can be reproduced in animal models (Roux et al., 2015; Wheatley et al., 2021).

To conclude, our results highlight that gene acquisition is more efficient than the accumulation of mutations to evolve multidrug resistance in *E. coli*. Although it is still unclear whether this finding may be generalized to most bacterial species, the lesser cost of horizontal transfer compared to mutational AMR stresses the need to monitor and control the diffusion of AMR plasmids as closely as the diffusion of resistant bacteria in the environment.

## Author contributions

MV contributed to the conception, design of the study, organization of the database and statistical analysis and wrote the first draft of the manuscript. All authors contributed to the article and approved the submitted version.

## Funding

This work was founded by the French National Research Agency, part of the AAPG 2020 programme (ANR-20-CE35-0012 to J-PR).

## Conflict of interest

The authors declare that the research was conducted in the absence of any commercial or financial relationships that could be construed as a potential conflict of interest.

## Publisher’s note

All claims expressed in this article are solely those of the authors and do not necessarily represent those of their affiliated organizations, or those of the publisher, the editors and the reviewers. Any product that may be evaluated in this article, or claim that may be made by its manufacturer, is not guaranteed or endorsed by the publisher.

## Supplementary material

The Supplementary material for this article can be found online at: https://www.frontiersin.org/articles/10.3389/fmicb.2023.1186920/full#supplementary-material
