# Supplementary material for: The fitness cost of horizontally transferred and mutational antimicrobial resistance in Escherichia coli
Source: Front Microbiol. 2023 Jun 30;14:1186920. doi: 10.3389/fmicb.2023.1186920 (PMC10348881; doi:10.3389/fmicb.2023.1186920)
Supplement: Supplementary file 1 [file Presentation_1.PDF]

Supplementary Material

The fitness cost of horizontally transferred and mutational antimicrobial resistance in *Escherichia coli*

Marie Vanacker, Natacha Lenuzza, Jean-Philippe Rasigade*

*** Correspondence:** jean-philippe.rasigade@univ-lyon1.fr

1 Supplementary Data

Data S1 corresponds to a excel files with in the first sheet, the list of all studies found in PubMed, and the criteria of non-selection. In the second sheet, there is the list of the 46 papers included in the meta-analysis with the methods of estimation of fitness used in the corresponding paper.

Data S2 corresponds to the data linked to ARGs and AMRs. In the first sheet, there is the list of the 151 ARGs observed in the whole dataset. In the second sheet, the presence/absence of the major AMR families in the 783 strains.

Data S3 had only one excel sheet, and corresponds to the all-strains data observed in all included papers, and the characteristics linked to analysis.

2 Supplementary Figures and Tables

2.1 Figures

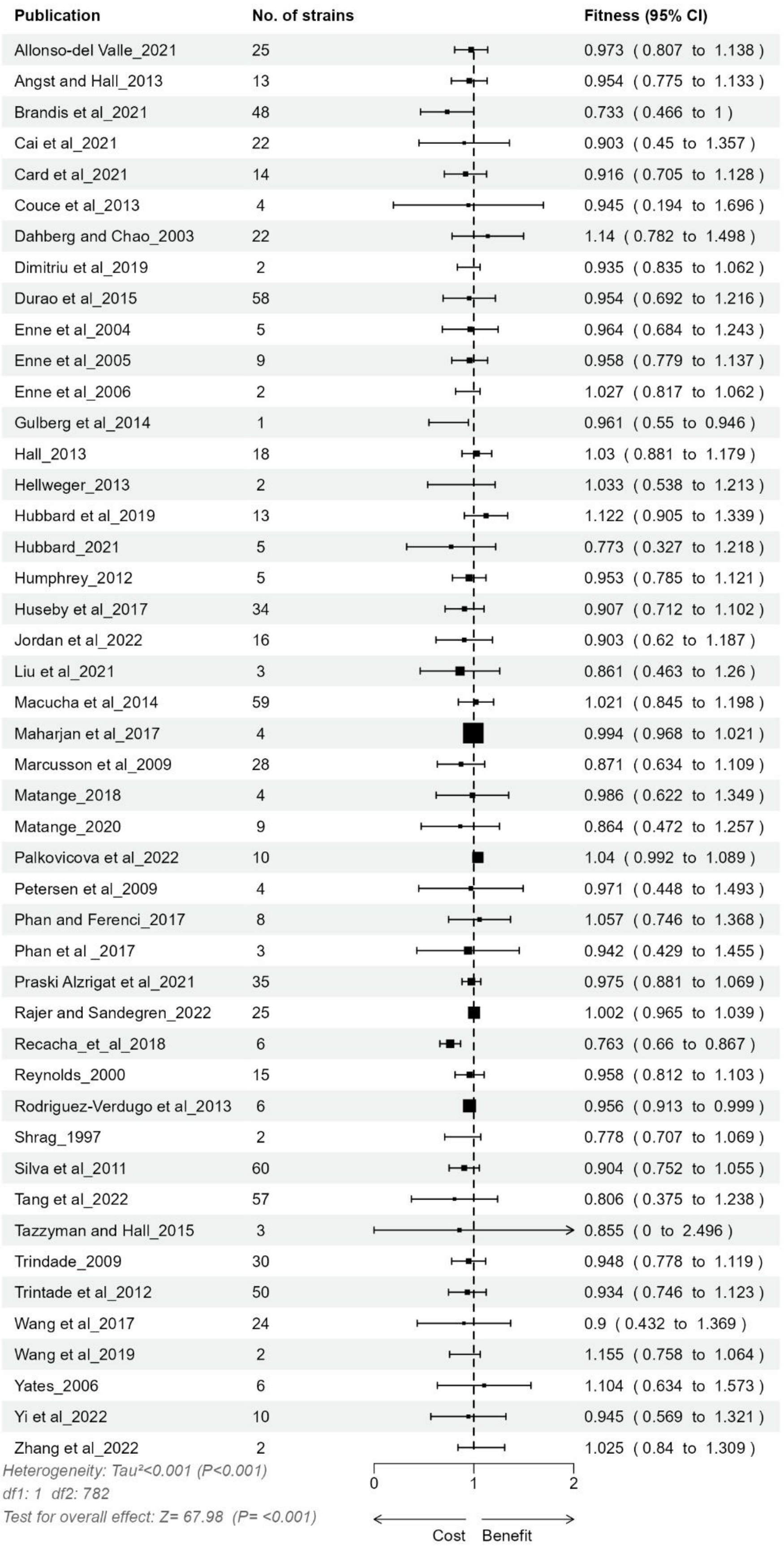
Publication No. of strains Fitness (95% CI)
Allonso-del Valle_2021 25 0.973 (0.807 to 1.138)
Angst and Hall_2013 13 0.954 (0.775 to 1.133)
Brandis et al_2021 48 0.733 (0.466 to 1)
Cai et al_2021 22 0.903 (0.45 to 1.357)
Card et al_2021 14 0.916 (0.705 to 1.128)
Couce et al_2013 4 0.945 (0.194 to 1.696)
Dahberg and Chao_2003 22 1.14 (0.782 to 1.498)
Dimitriu et al_2019 2 0.935 (0.835 to 1.062)
Durao et al_2015 58 0.954 (0.692 to 1.216)
Enne et al_2004 5 0.964 (0.684 to 1.243)
Enne et al_2005 9 0.958 (0.779 to 1.137)
Enne et al_2006 2 1.027 (0.817 to 1.062)
Gulberg et al_2014 1 0.961 (0.55 to 0.946)
Hall_2013 18 1.03 (0.881 to 1.179)
Hellweger_2013 2 1.033 (0.538 to 1.213)
Hubbard et al_2019 13 1.122 (0.905 to 1.339)
Hubbard_2021 5 0.773 (0.327 to 1.218)
Humphrey_2012 5 0.953 (0.785 to 1.121)
Huseby et al_2017 34 0.907 (0.712 to 1.102)
Jordan et al_2022 16 0.903 (0.62 to 1.187)
Liu et al_2021 3 0.861 (0.463 to 1.26)
Macucha et al_2014 59 1.021 (0.845 to 1.198)
Maharjan et al_2017 4 0.994 (0.968 to 1.021)
Marcusson et al_2009 28 0.871 (0.634 to 1.109)
Matange_2018 4 0.986 (0.622 to 1.349)
Matange_2020 9 0.864 (0.472 to 1.257)
Palkovicova et al_2022 10 1.04 (0.992 to 1.089)
Petersen et al_2009 4 0.971 (0.448 to 1.493)
Phan and Ferenci_2017 8 1.057 (0.746 to 1.368)
Phan et al _2017 3 0.942 (0.429 to 1.455)
Praski Alzrigat et al_2021 35 0.975 (0.881 to 1.069)
Rajer and Sandegren_2022 25 1.002 (0.965 to 1.039)
Recacha_et_al_2018 6 0.763 (0.66 to 0.867)
Reynolds_2000 15 0.958 (0.812 to 1.103)
Rodriguez-Verdugo et al_2013 6 0.956 (0.913 to 0.999)
Shrag_1997 2 0.778 (0.707 to 1.069)
Silva et al_2011 60 0.904 (0.752 to 1.055)
Tang et al_2022 57 0.806 (0.375 to 1.238)
Tazzyman and Hall_2015 3 0.855 (0 to 2.496)
Trindade_2009 30 0.948 (0.778 to 1.119)
Trintade et al_2012 50 0.934 (0.746 to 1.123)
Wang et al_2017 24 0.9 (0.432 to 1.369)
Wang et al_2019 2 1.155 (0.758 to 1.064)
Yates_2006 6 1.104 (0.634 to 1.573)
Yi et al_2022 10 0.945 (0.569 to 1.321)
Zhang et al_2022 2 1.025 (0.84 to 1.309)
Heterogeneity: Tau²<0.001 (P<0.001)
df1: 1 df2: 782
Test for overall effect: Z= 67.98 (P= <0.001)
0
1
2
Cost
Benefit

Supplementary Figure S1. Results showing significant difference between the 46 papers included in the database (P-value <0.001 for overall effect). Each vertical line represents the confidence interval with the effect size of the group denoted by the square. The area of the square is proportional to the corresponding group weight in the overall effect.

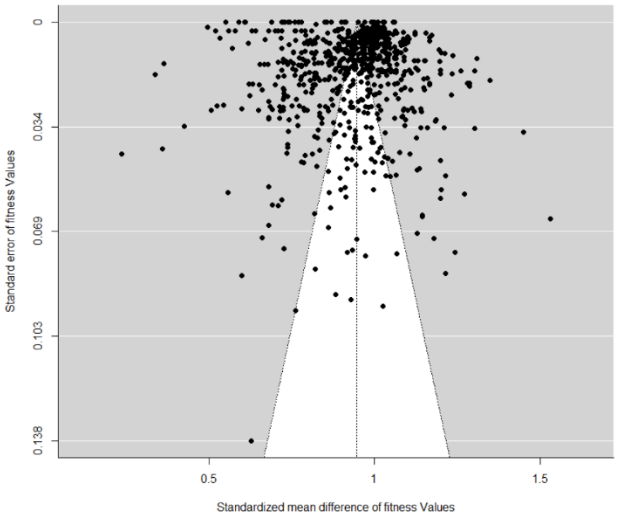

Supplementary Figure S2. Funnel plot results showing the effect size of each study (expressed as the standardized mean difference of fitness values) on the x-axis, and the standard error of the fitness values (from large to small) on the y-axis. The vertical line in the middle of the funnel shows the average effect size with 95% confidence interval. The funnel plot showed high heterogeneity of fitness values in the whole dataset.

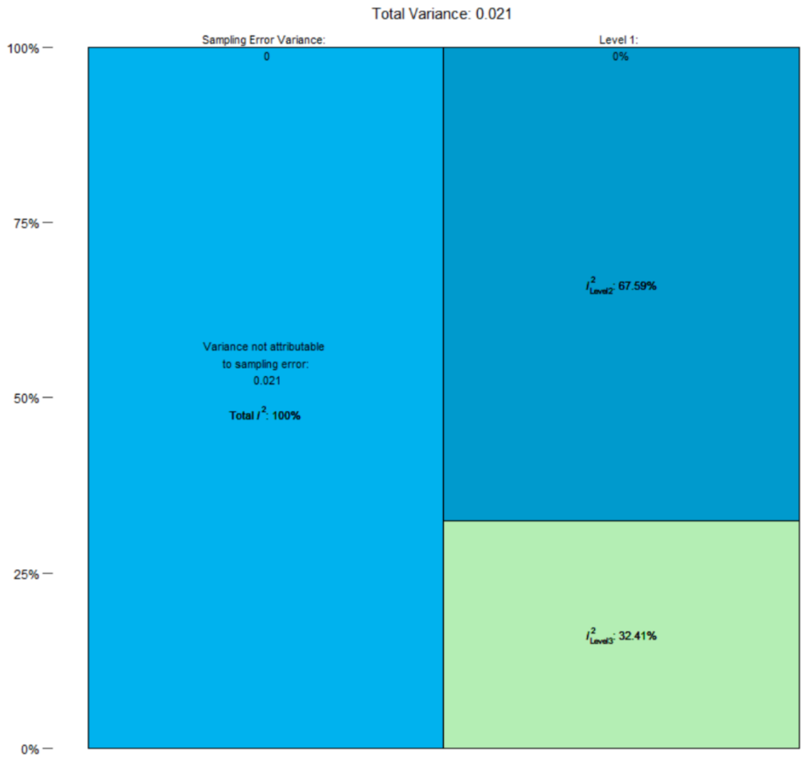

Supplementary Figure S3. Results showing the variance decomposition of the multi-level analysis. 32% of the overall variance can be attributed to level 3 (inter-study variance), 68% to level 2 (intra-study variance) and 0% to the level 1

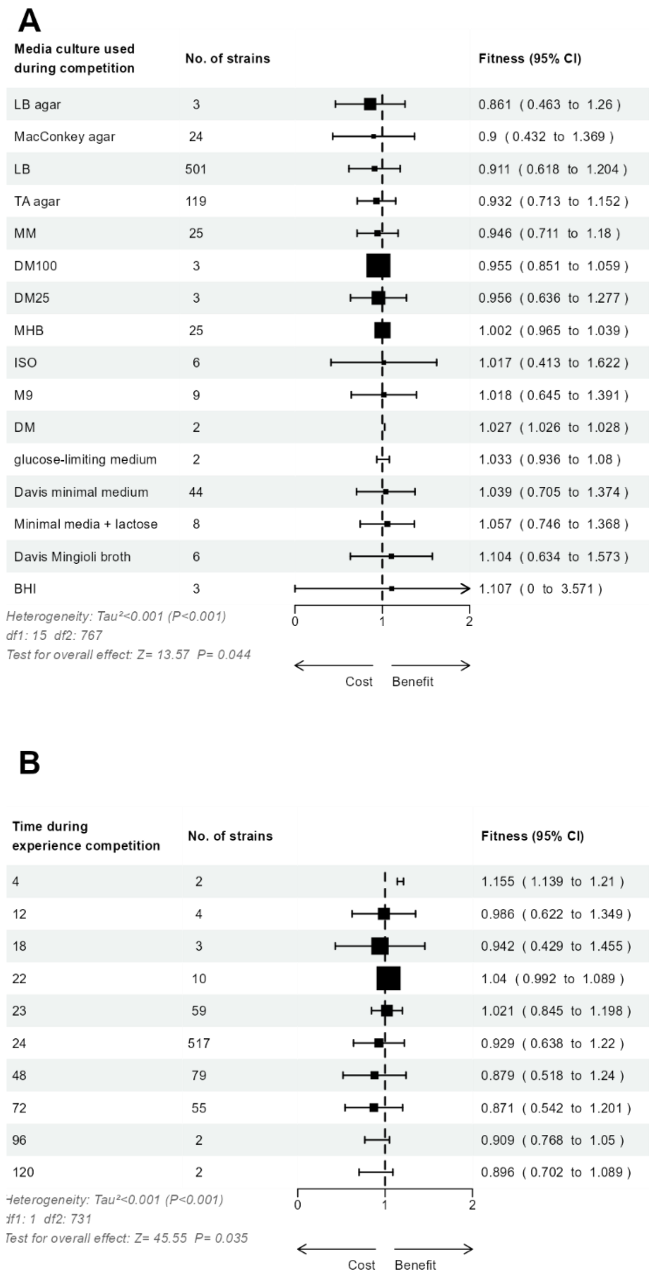

Supplementary Figure S4. Forest plot of the meta-analysis following the media culture used during the laboratory competition experiments (A) and the time of the experiments (B). Each vertical line represents the confidence interval with the effect size of the group denoted by the square. The area of the square is proportional to the corresponding group weight in the overall effect. The meta-analyses of both proxies showed significant differences for the overall effect (P-values of 0.044 and 0.035 respectively).

A

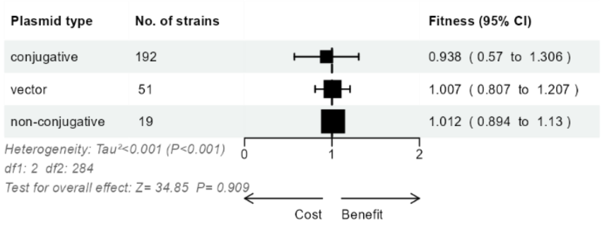

B

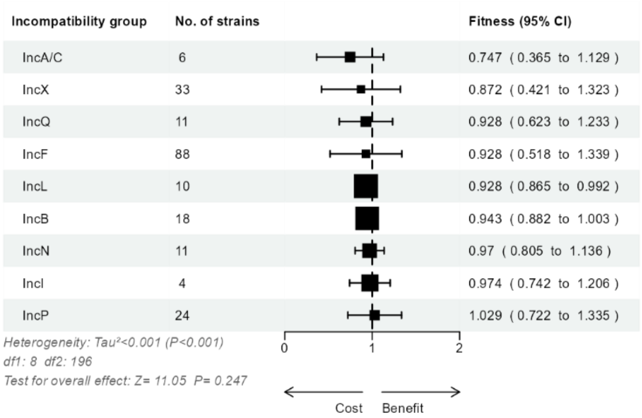

Supplementary Figure S5. Forest plot results of meta-analysis in resistant strains having acquired resistance with transferable ARGs by one plasmid in the host (all strains combined). Effects of the plasmid type (A and the incompatibility group of the plasmid. Results showed no significant effect (P-value of the overall effect >0.05 in the both cases). Each vertical line represents the confidence interval with the effect size of the group denoted by the square. The area of the square is proportional to the corresponding group weight in the overall effect.

2.2 Tables

Table S1. Percentage of E. coli data and other species in the meta-analyses of Melnyk et al. (2015) and Vogwill et al. (2015).

**Species**	**Melnyk et al. 2015**		**Vogwill et al. 2015**	
	**Mutation Number**	**% Mutation**	**Publication Number**	**% Publication**
Acinetobacter baylyi			1	1.3
Bacillus subtilis			1	1.3
Borrelia burgdorferi	2	1.1	1	1.3
Campylobactor jejuni	10	5.6	3	3.9
Chlamydia psittaci			2	2.6
Enterococcus faecium	9	5.0	3	3.9
**Escherichia coli**	**51**	**28.5**	**21**	**27.3**
Haemophilus influenzae			1	1.3
Mycobacterium abscessus			1	1.3
Mycobacterium smegmatis	20	11.2	2	2.6
Mycobacterium tuberculosis	12	6.7	4	5.2
Neisseria gonorrhoeae			1	1.3
Pseudomonas aeruginosa			4	5.2
Pseudomonas putida			1	1.3
Salmonella enterica			5	6.5
Salmonella typhimurium			4	5.2
Salmonella typhoid			2	2.6
Staphyloccus aureus	62	34.6	13	16.9
Staphyloccus epidermidis			1	1.3
Streptococcus gordonii			1	1.3
Streptococcus pneumoniae	13	7.3	3	3.9
Various			2	2.6
**Total**	**179**	**100**	**77**	**100**
